# Feasibility of bone marrow sparing volumetric modulated arc therapy to spare active bone marrow in cervical and vaginal cancer patients: a retrospective dosimetric analysis

**DOI:** 10.1002/jmrs.529

**Published:** 2021-07-19

**Authors:** Michaela Beavan, Kylie Dundas, Felicity Hudson, Yolanda Surjan, Annie Lau, Shrikant Deshpande, Karen Lim, Viet Do

**Affiliations:** ^1^ Medical Radiation Science (MRS) School of Health Sciences The University of Newcastle Callaghan New South Wales Australia; ^2^ Liverpool and Macarthur Cancer Therapy Centres South West Sydney Local Health District Liverpool New South Wales Australia; ^3^ Ingham Institute of Applied Medical Research Liverpool, Sydney New South Wales Australia; ^4^ South Western Sydney Clinical School University of New South Wales Sydney New South Wales Australia

**Keywords:** active bone marrow, bone marrow sparing, gynaecological cancer, volumetric modulated arc therapy

## Abstract

**Introduction:**

Chemoradiotherapy (CRT) is the standard treatment for locally advanced cervical and vaginal cancer. It is associated with high haematological toxicity (HT) that can lead to treatment interruptions and cancelled chemotherapy cycles, reducing the potential effectiveness of this regimen. Bone marrow sparing (BMS) utilising volumetric modulated arc therapy (VMAT) is one method to reduce dose to the active bone marrow (ABM) so that HT rates are reduced. The aim of this paper was to assess whether BMS‐VMAT can effectively spare the ABM whilst maintaining clinically acceptable target and organ‐at‐risk (OAR) doses.

**Methods:**

Twenty gynaecological cancer patients treated with definitive CRT at the Liverpool/Macarthur Cancer Therapy centres between 2015 and 2020 were retrospectively included. ABM was delineated based on fluorodeoxyglucose positron emission tomography (FDG‐PET) imaging. Weekly blood tests and ABM dose parameters at the V10Gy, V20Gy, V30Gy, V40Gy and Dmean were assessed on original plans for any potential correlation with grade 2+ HT. Replanned with VMAT for BMS, various dose parameters were compared with the original plan to assess for any significant differences.

**Results:**

Active bone marrow doses were significantly reduced (*P* < 0.001 for all parameters) in BMS‐VMAT plans, and significant improvements in target and OAR coverage were found compared with the original plans. Compared with VMAT only, target and OARs were comparable. No significant correlations between HT and ABM doses were found.

**Conclusion:**

Bone marrow sparing volumetric modulated arc therapy can significantly reduce dose to the active bone marrow whilst maintaining acceptable target and OAR doses. Future prospective trials are needed to evaluate the clinical impact of BMS on toxicity and compliance.

## Graphical abstract

Bone marrow sparing volumetric modulated arc therapy (BMS‐VMAT) is a recent technique aimed at reducing haematological toxicity in patients with cervical and vaginal cancer undergoing chemoradiotherapy that could potentially result in treatment interruptions. Our retrospective study found that significant dosimetric sparing in active areas of bone marrow could be achieved, whilst maintaining clinically acceptable target and organ‐at‐risk doses, comparable to VMAT where BMS was not considered.

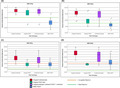

## Introduction

In Australia, gynaecological cancers account for 9% of all reported cancers in women. Of this, cervical and vaginal cancer account for 17.2% and 1.5–2% of all gynaecological cancers, respectively.^1^ Radical concurrent chemoradiotherapy (CRT) is the current standard of treatment of locally advanced cervical and vaginal cancer, improving local control, and reducing local failure and distant metastatic rates compared with radiotherapy alone.^2^, ^3^ Despite the benefits of current treatment, the 5‐year survival rates for cervical cancer and vaginal cancer are 73.5% and 45%, respectively.^1^ The myelosuppressive effects of combination CRT result in a range of haematological toxicities (HT) that can lead to cancelled chemotherapy cycles and delays in both chemotherapy and radiotherapy treatments.^4^, ^5^ In one study, chemotherapy was delayed in 35% or cancelled for at least one cycle in 44% of patients.^4^ Delays in treatment are problematic, and a recent study found that 21% of patients exceed 50 days of overall treatment time, worsening local control.^6^

Bone marrow is the primary site of haematopoiesis, and in adults, >50% of proliferating bone marrow is located within the pelvis, including the lumbar spine.^7^ Multiple studies have suggested that there is a correlation between HT and volume of bone marrow irradiated, however, the specific dose–volume objectives between these studies are inconsistent.^7^, ^8^, ^9^, ^10^, ^11^, ^12^ Bone marrow sparing (BMS) techniques aim to reduce HT associated with CRT treatment, primarily focussing on pelvic sites.^8^, ^13^, ^14^, ^15^, ^16^, ^17^ Proximity of the bone marrow to the planning target volume (PTV) can be technically challenging; however, with the use of techniques such as volumetric modulated arc therapy (VMAT), BMS techniques may be plausible in achieving bone marrow reductions.

There are few studies investigating BMS techniques for cervical cancer with no widely accepted protocols or guidelines. Further uncertainty is enhanced by the inconsistency of bone marrow delineation across studies.^8^, ^13^, ^14^, ^15^, ^16^, ^17^ Fluorodeoxyglucose positron emission tomography (FDG‐PET) has been demonstrated to be superior to computerised tomography (CT), which cannot distinguish between active and inactive areas, in defining haematopoietically active bone marrow (ABM) for HT predictions.^9^, ^16^, ^18^, ^19^ Its use in other retrospective studies is limited, with BMS‐VMAT (excluding intensity modulated radiotherapy (IMRT)) studies delineating bone marrow based on CT only. This overestimates the bone marrow contours, leading to over‐optimisation of these contours and constraining the optimisation process.^13^, ^17^ Prospective trials in this area are few, with Mell et al. conducting the only trial investigating FDG‐PET‐defined ABM specifically in cervical cancer (*n* = 83) reporting reductions in the rate of HT.^14^ Despite the differences in ABM delineation, all studies demonstrated, dosimetrically, that dose to the ABM can be reduced without compromise of target coverage or organs at risk (OAR) dose.

The aim of this retrospective study was to assess the feasibility of BMS‐VMAT in reducing ABM dose without compromising clinically acceptable plans in patients with cervical and vaginal cancer. This study will assist in the protocol development for a future prospective trial.

## Method

This study has received ethics approval from the South Western Sydney Local Health District Human Research Ethics Committee, HREC reference: HREC/16/LPOOL/603.

### Patient selection

Twenty consecutive patients who completed gynaecological CRT between December 2015 and February 2020 at Liverpool/Macarthur Cancer Therapy Centres were retrospectively analysed. Patients were included if they were classified as FIGO (International Federation of Gynecology and Obstetrics) stage Ib‐IVa cervical or endocervical cancer, or stage I–II vaginal cancer, treated definitively, had accessible staging PET scans and had weekly blood results available for analysis.

### Chemotherapy

Cisplatin was prescribed to 40 mg/m^2^ for 5–6 cycles once weekly or carboplatin prescribed to area under the curve (AUC) 2.

### External beam radiation therapy

#### Simulation

Patients were simulated on a Philips (Koninklijke Philips N.V., Amsterdam, The Netherlands) big bore CT scanner with 2‐mm slice thickness. Positioning was standard supine with full bladder as per departmental technique. Empty bladder magnetic resonance images (MRIs) were obtained in‐house on a wide‐bore 3 Tesla Siemens Skyra (Magnetom, Erlangen, Germany) MRI simulator. MRIs were fused with the primary CT for target delineation for internal target volume (ITV) creation.

#### *Other* *imaging*

All patients received staging FDG‐PET scans on either a GE (General Electric Company, Boston, Massachusetts) Discovery 710 PET/CT (*n* = 15) or GE Discovery MI Digital PET/CT (*n* = 5). Patients were scanned 60 min (±10%) after injection of FDG, dosed to 4.1 MBq/kg (±10%).

#### *Planning* *and contouring*

**Figure 1:**
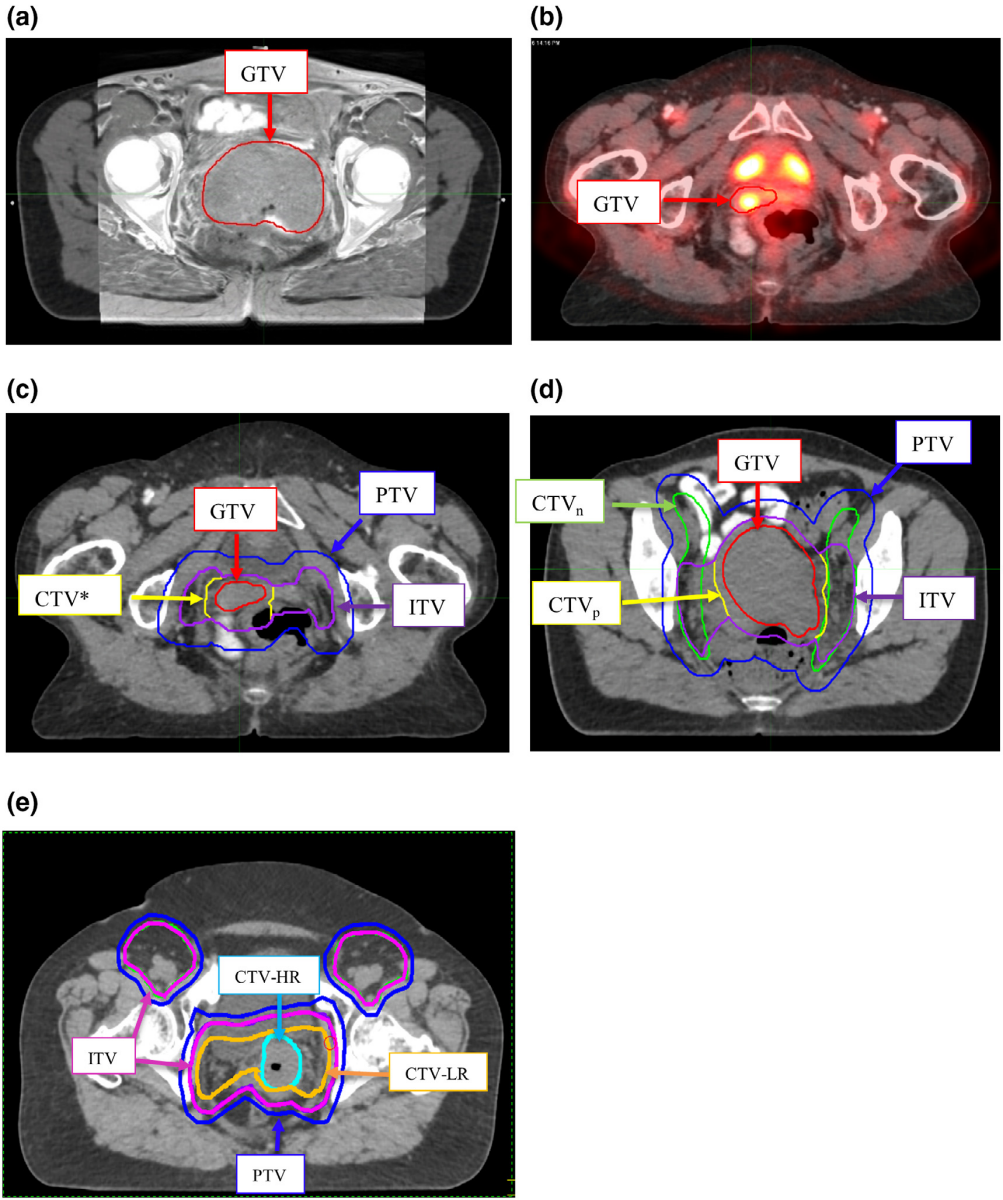
Target contouring. (A) MRI/CT delineation of GTV in cervical example. (B) PET/CT delineation of GTV in vaginal example. PET and/or MRI may be used in both cervical and vaginal cancer cases. (C) Target contours in vaginal case. CTV = GTV + 0.7 cm. ITV = GTV + 0.5 cm. PTV = CTV/ITV + 1 cm. (D) Target contours in standard cervix case. CTV = (seroma + 0.5 cm) + (GTV + 0.5 cm for VMAT / +1 cm for conformal). PTV = ITV/CTV + 1 cm. (E) Target contours in EMBRACE‐II cervix case. PTV = ITV + 0.5 cm. CT = computerised tomography, CTV = clinical target volume, CTV‐HR = high‐risk CTV, CTV‐LR = low‐risk CTV, CTVn = nodal CTV volume, CTVp = primary CTV volume, GTV = gross tumour volume, ITV = internal target volume, MRI = magnetic resonance imaging, PET = positron emission tomography, PTV = planning target volume, VMAT = volumetric modulated arc therapy.

Organ‐at‐risks were contoured as per Radiation Therapy Oncology Group (RTOG) normal tissue guidelines.^20^ Process of target contour generation is shown in Figure 1 and was dependent on whether the plan was to be created with a four‐field conformal, VMAT or EMBRACE II trial techniques. At initial planning, all target and OAR contours were independently audited by a second radiation oncologist.

### Haematologic toxicity

Toxicity results were retrospectively extracted from weekly blood test reports. Results were analysed and graded according to the Common Terminology Criteria for Adverse Events (CTCAE) v5.0.^21^ Neutrophil, white blood cells, platelet and haemoglobin nadirs were recorded, and the greatest toxicity in any of these categories was used to define overall HT. Analysis based on overall number of grade 2 events (< grade 2 vs ≥ grade 2) compared with the ABM dose levels V10Gy, V20Gy, V30Gy, and V40Gy in original plans was conducted to test for any correlations.

### Bone marrow sparing replanning

#### *Bone* *marrow contouring*

Contouring of the total bone marrow (TBM) and ABM was completed within MiM (MiM Software Inc., Cleveland, Ohio), version 6.9.5. All previous contours were imported with the original plan. In patients where an available PET scan was not originally fused (*n* = 5), images were retrieved from Picture Archiving and Communication System and rigidly fused with the planning CT in MiM, matching to the bony anatomy. The PET/CT was used to manually contour the TBM, defined as the external bony contours from the superior level of L5 to the inferior level of the lesser trochanter of the femur. ABM was then defined on PET/CT as the region within the TBM that had a standard uptake value (SUV) ≥ the mean uptake in the TBM, described by previous authors.^19^, ^22^

#### *Planning* *parameters*

Patient cases were replanned with 6MV VMAT with two full arcs in Pinnacle (Koninklijke Philips N.V., Amsterdam, Netherlands), version 9.1 (*n* = 14) and 16.02 (*n* = 6), depending on the version they were originally planned. The target volume and OAR objectives (excluding ABM) follow ICRU 83 and EMBRACE recommendations, shown in Table 1.^6^, ^23^ ABM objectives were determined from previously published studies.^7^, ^8^, ^9^, ^10^, ^11^, ^12^

**Table 1:** Planning objectives.

Structure	Ideal	Acceptable	Non‐Compliant
CTV/ITV	D98 = 100% D95 = 100%	D98 ≥ 97% of TD D95 ≥ 98% of TD Dmin ≥ 95%^1^ V95 ≥ 100% V100 ≥ 97%^2^	D98 < 97% D95 < 98% Dmin < 95%^1^ V95 < 95% V100 < 97% ^2^
PTV	D98 ≥ 95% D95 ≥ 97%	D95 ≥ 95% of TD V95 ≥ 95%	D 95 < 95% V95 < 95%
Overall plan	Point Max < 107% D50 ≤ TD + 2Gy	D2 < 107%	D2 > 107%
Active bone marrow	V10Gy < 90%^7^, ^11^ V20Gy < 75%^10^, ^11^ V30Gy < 46.5%^10^ V40Gy < 23%^9^, ^10^	V30Gy < 60%^8^ V40Gy < 37%^12^	If exceeding objective OR originally accepted constraint by 2% (whichever is greater)
Small bowel (TD = 45Gy)	V40Gy < 100 cm^3^		
	V30Gy < 350 cm^3^		
	Point max ≤ TD	Points max < 105%	
Small bowel (TD = 50.4Gy)	V40Gy < 250 cm^3^		
	V30Gy < 500 cm^3^		
	Point max ≤ TD	Point max < 103%	
Rectum	V40Gy < 85%	V40Gy < 90%	
	V30Gy < 95%	V30Gy < 100%	
	Point max ≤ 102%	Point max < 105%	
Iliac crests	V30Gy < 50%	V30Gy < 55%	
	V40Gy < 35%	V40Gy < 40%	
	V50Gy < 5%	V50Gy < 8%	
Femoral heads	V30Gy < 15%	V30Gy < 20%	
	Point Max < 47.5Gy	Point Max < 50Gy	
External genitalia	V20Gy < 50%	V20Gy < 55%	
	V30Gy < 35%	V30Gy < 40%	
	V40Gy < 5%	V40Gy < 8%	
Bladder	V40Gy < 60%	V40Gy < 75%	
	V30Gy < 75%	V30Gy < 85%	
	Point Max < 102%		
Spinal cord/cauda equina	Point Max < 48Gy		
Kidney	Mean < 15Gy	Mean < 18Gy	

CTV = clinical target volume, Dmean = mean dose, Dmin = minimum dose, Dx = x% of structure receiving % of TD, GTV = gross tumour volume,Gy = gray, ITV = internal target volume, PTV = planning target volume, TD = total dose, Vx = x% of dose covering % of structure.

^1^ For EMBRACE II participant ONLY.

^2^ Not considered in EMBRACE II participant.

ABM objective was given a relatively low priority, in comparison with target and standard OAR structures, to ensure current treatment standards were maintained. Where OAR coverage could not be achieved due to compromised target coverage, OAR doses were reduced to as low as achievable and must not exceed originally accepted constraint by 2%. All plans were reviewed by a senior gynaecological planner to determine its clinical acceptability.

### Statistical analysis

Statistical analysis was conducted using SPSS statistics software (IBM Corp., SPSS Statistics for Windows, Version 26.0; Armonk, New York, 2019). Fisher’s exact test was used to compare rates of HT grades and ABM receiving V10Gy, V20Gy, V30Gy and V40Gy levels (dichotomised at the median). The paired sample sign test was used for comparison of the volume of ABM receiving 10Gy, 20Gy, 30Gy and 40Gy in BMS and original plans because data were not normally distributed, and distribution of differences was asymmetrical. Comparisons in clinical target volume (CTV), ITV, PTV and OAR doses were also conducted using this test at their respective dose levels, shown in Table 1. This test was also used when original plans were separated into VMAT and conformal, investigating the same objectives. Statistical significance was assessed at a significance level of 5% (i.e. *P* < 0.05).

## Results

### *Patient* *and treatment characteristics*

The patient and treatment characteristics are shown in Table 2. Three of six participants did not complete five cycles of chemotherapy due to HT. One participant was able to complete treatment with blood transfusion.

**Table 2:** Patient characteristics and treatment techniques.

		*N* (%)
Patient characteristics		
Age (years)	30–39	1 (5)
	40–49	7 (35)
	50–59	4 (20)
	60–69	3 (15)
	70–79	5 (25)
Site	Cervix	15 (75)
	Endocervix	3 (15)
	Vagina	2 (10)
FIGO Stage	IB	3 (15)
	II	2 (10)
	IIA	3 (15)
	IIB	5 (25)
	IIIB	6 (30)
	IVA	1 (5)
BMI	<18.5 (underweight)	1 (5)
	18.5–24.9 (healthy)	5 (25)
	25–29.9 (overweight)	7 (35)
	≥30 (obese)	7 (35)
Treatment characteristics		
Radiotherapy prescription	45Gy/25fx	14 (70)
	50.4Gy/28fx	6 (30)
Original radiotherapy technique	Conformal	14 (70)
	VMAT	6 (30)
Chemotherapy drug	Cisplatin	18 (90)
	Carboplatin ^1^, ^2^	2 (10)
Chemotherapy compliance	5 cycles	14 (70)
	<5 cycles	6 (30)
Haematological toxicity outcomes		
Highest HT grade	<Grade 2	6 (30)
	≥Grade 2	14 (70)
Leukopenia	<Grade 2	9 (45)
	≥Grade 2	11 (55)
Neutropenia	<Grade 2	11 (55)
	≥Grade 2	9 (45)
Anaemia	<Grade 2	8 (40)
	≥Grade 2	12 (60)
Platelets	<Grade 2	16 (80)
	≥Grade 2	4 (20)

BMI = body mass index, FIGO = International Federation ofGynaecology and Obstetrics, Fx = fraction, Gy = gray, HT = haematologic toxicity, VMAT = volumetric modulated arc therapy.

^1^ Treated with carboplatin instead of cisplatin due to hearing impairment.

^2^ Patient changed from cisplatin to carboplatin because of worsened glomerular filtration rate.

### *Correlation* *between bone marrow doses and haematological toxicity*

The median parameters at ABM V10Gy, V20Gy, V30Gy, V40Gy and Dmean for original plans were 97.5%, 90.5%, 71%, 54% and 36.2Gy, respectively. Two outliers were removed (*n* = 18) for analysis. One participant had significantly lower ABM doses due to the vaginal PTV being inferiorly located in comparison with cervix primaries, and it did not encompass nodal chains above the sacrum. Another participant had treatment discontinued after 15 fractions due to severe gastric toxicity. No correlation with grade 2+ HT was found at any dose level.

### *Bone* *marrow sparing comparisons*

Results of the ABM, planning target and OAR doses for both original and BMS plans are shown in Table 3. Only significant target objectives and relevant dose‐limiting OARs are presented; however, additional structures are shown in Table S1. Target coverage was significantly improved at all objectives in comparison with the original plans. When conducting a subgroup analysis comparing the original (non‐BMS) VMAT plans with BMS‐VMAT, BMS‐VMAT target doses were non‐significant in comparison with original VMAT plans. The PTV D95 was significantly worse in BMS‐VMAT plans (44.12Gy) compared with the original VMAT (44.57Gy). Similarly, for the OARs, significant improvements were noted in BMS‐VMAT plans compared with combined original plans, but not significant in comparison with original VMAT only.

**Table 3:** Median values of target and OAR doses between original and BMS‐VMAT plans.

Structures		Planning Median				Significance		
		Original			BMS			
Name	Constraint	Conformal (*n* = 14) (IQR)	VMAT (*n* = 6) (IQR)	Total (*n* = 20) (IQR)	VMAT (*n* = 20) (IQR)	Original combined vs BMS	Original conformal vs BMS	Original VMAT vs BMS
ABM	V10Gy	97.97% (2.79)	93.86% (7.95)	96.1% (4.38)	85.08% (4.08)	*P* < 0.001*	*P* < 0.001*	*P* = 0.031*
	V20Gy	92.16% (5.55)	78.27% (11.30)	90.27% (11.22)	72.54% (4.55)	*P* < 0.001*	*P* < 0.001*	*P* = 0.031*
	V30Gy	72.96% (11.78)	58.61% (9.54)	69.80% (14.62)	57.51% (8.59)	*P* < 0.001*	*P* < 0.001*	*P* = 0.219
	V40Gy	55.54% (9.06)	36.46% (5.62)	54.35% (21.58)	38.09% (8.94)	*P* < 0.001*	*P* < 0.001*	*P* = 0.219
CTV	D95%	45.40Gy (4.88)	45.29Gy (4.42)	45.29Gy (4.75)	45.41Gy (5.74)	*P* = 0.021*	*P* = 0.013*	*P* = 0.219
	D98%	45.14Gy (5.10)	45.06Gy (4.59)	45.11Gy (4.95)	45.27Gy (5.49)	*P* = 0.013*	*P* = 0.013*	*P* = 0.125
	V100%	96.1% (8.02)	98.48% (2.67)	97.19% (7.75)	99.70% (0.59)	*P* < 0.001*	*P* = 0.002*	*P* = 0.219
ITV	D95%	45.15Gy (3.45)	45.20Gy (0.50)	45.19Gy (0.77)	45.51Gy (0.26)	*P* = 0.002*	*P* = 0.109	*P* = 0.700
	D98%	44.98Gy (3.63)	44.99Gy (0.51)	44.99Gy (1.11)	45.41Gy (0.31)	*P* = 0.002*	*P* = 0.021*	*P* = 0.125
	V100%	95.51% (11.39)	97.90% (4.00)	96.58% (5.81)	99.97% (0.38)	*P* = 0.002*	*P* = 0.021*	*P* = 0.289
PTV	D95%	44.55Gy (5.13)	44.72Gy (2.15)	44.57Gy (4.96)	44.12Gy (4.97)	*P* < 0.001*	*P* = 0.021*	*P* = 0.004*
	D98%	44.06Gy (4.90)	44.41Gy (2.07)	44.16Gy (4.80)	44.85Gy (4.95)	*P* = 0.001*	*P* = 0.021*	*P* = 0.039*
Overall Plan	D2%	105.13% (1.11)	104.71% (1.60)	104.80% (1.02)	103.78% (0.48)	*P* < 0.001*	*P* = 0.002*	*P* = 0.125
Small bowel	V30Gy	808.74cc (502.44)	612.41cc (438.85)	720.71cc (467.69)	639.30cc (368.23)	*P* < 0.001*	*P* < 0.001*	*P* = 0.219
	V40Gy	486.69cc (269.09)	375.82cc (367.73)	457.62cc (255.88)	359.91cc (244.22)	*P* < 0.001*	*P* < 0.001*	*P* = 0.219
Rectum	V30Gy	97.65% (5.27)	83.69% (22.74)	97.12% (10.03)	94.77% (11.78)	*P* = 0.021*	*P* = 0.065	*P* = 0.375
	V40Gy	93.72% (11.31)	67.61% (27.37)	90.13% (14.34)	80.53% (23.49)	*P* < 0.001*	*P* < 0.001*	*P* = 0.688
Bladder	V30Gy	100% (2.98)	91.10% (19.87)	98.70% (4.94)	88.24% (20.56)	*P* < 0.001*	*P* = 0.001*	*P* = 0.031*
	V40Gy	95.59% (9.45)	62.44% (32.61)	92.03% (21.37)	70.36% (33.57)	*P* = 0.001*	*P* < 0.001*	*P* = 0.688

ABM = active bone marrow, BMS = bone marrow sparing, CTV = clinical target volume, Dx = x% of structure receiving % of TD, Gy = gray, IQR = interquartile range, ITV = internal target volume, LT = left, PTV = planning target volume, RT = right, SD = standard deviation, VMAT = volumetric modulated arc therapy, Vx = x% of dose covering % of structure.

\x90* Indicates statistical significance.

**Figure 2:**
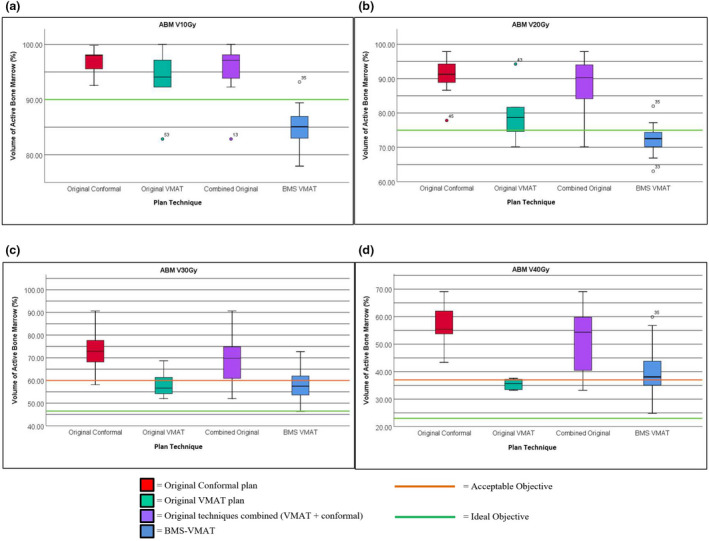
Boxplot of ABM doses for original and BMS plans. (A) Volume of bone marrow receiving 10Gy in original and BMS planning techniques. (B) Volume of bone marrow receiving 20Gy in original and BMS planning techniques. (C) Volume of bone marrow receiving 30Gy in original and BMS planning techniques. (D) Volume of bone marrow receiving 40Gy in original and BMS planning techniques. ABM = active bone marrow, BMS = bone marrow sparing,Gy = gray, VMAT = volumetric modulated arc therapy.

Figure 2 highlights ABM doses for V10‐40Gy, including their relation to ideal and acceptable (where applicable) objectives. In the original VMAT plans, ABM objectives were compliant (defined as achieving either ideal or acceptable dose objectives) in 16.7% and 33.3% of cases for the V10‐20Gy, and 50% for both V30‐40Gy. In the original conformal plans, compliance for the ABM V10‐20Gy and V40Gy was 0%, and 7.2% for the V30Gy.

BMS‐VMAT plans were compliant in 95%, 80%, 90% and 40% of cases for the ABM V10Gy, V20Gy, V30Gy, and V40Gy, respectively. When the originally planned technique was VMAT, 100% of plans met the ABM V10Gy‐30Gy, and 83.3% met the V40Gy. When the originally planned technique was conformal, ABM objectives met 92.9%, 71.4%, 85.7% and 21.4% for V10‐40Gy, respectively. Of the compliant BMS‐VMAT V30‐40Gy objectives, none met the ideal criteria (Table S2).

## Discussion

### *Haematological* *toxicity*

This analysis found no correlation between ABM dose parameters and HT, contradictory to current literature. Doses to the ABM were not a previous consideration in planning, resulting in over‐irradiation of this structure at all dose levels indicated by our high median ABM values, negating the potential for any sparing effect. This limitation was also reported by Klopp et al. when they analysed the ABM V10Gy parameter.^12^ This, coupled with our small sample size, precludes our ability to test for significance.

Literature investigating ABM dose objectives demonstrates that a correlation exists between HT and certain ABM dose levels, albeit there is little consistency in the specific cut‐off points.^7^, ^8^, ^9^, ^10^, ^11^, ^12^ Previous retrospective studies utilising CT‐defined bone marrow suggest that V10Gy and V20Gy are predictive of acute HT.^7^, ^11^ Mell et al. reported that rates of grade 2+ neutropenia and leukopenia were reduced from 73.1% to 11.1% and 31.6% to 5.6%, respectively, when the V10Gy<90% and V20Gy < 75%.^7^ Similarly, Rose et al. found that rates of grade 3+ HT are reduced by 50% when V10Gy < 95% and V20Gy < 76%.^11^ In contrast, a prospective Klopp et al. study found that only the V40Gy < 37% could reduce rates of grade 2+ HT from 75% to 40%, not the V10‐20Gy.^12^ These studies are limited by their bone marrow delineation methods using CT only, and therefore, whether these objectives are applicable to functionally defined ABM cannot be determined.^14^, ^16^

More recent studies have focussed on correlations between HT and functionally defined bone marrow; however, results are also inconsistent. In these studies, there is a heterogeneity in image modality used for ABM delineation. In a 2019 prospective study utilising technetium‐99m sulphur colloid single‐photon emission tomography (SPECT)‐defined ABM in 39 patients with cervical cancer, ABM V30Gy < 46.5% and V40Gy < 23.5% reduced the rates of grade 3+ HT.^10^ Supporting the V40Gy dose objective, David et al. found that when V40Gy < 20–25% in FDG‐PET‐defined ABM, rates of grade 2+ toxicity could be reduced in anal cancers, which correlated with pre‐ and post‐PET scans.^9^ Accurate identification of ABM is essential to improve the optimisation process; however, there is a lack of consensus on the optimal imaging modalities. FDG‐PET is more favourable because it is standardly used in clinical staging and is more common in comparison with alternative functional imaging modalities such as SPECT and 18F‐fluorothymidine PET.^10^, ^24^

Determining which ABM dose objectives should be prioritised is difficult due to the lack of consensus. The V10‐20Gy has been optimised in prospective research, which have demonstrated reductions in HT.^14^, ^16^ We found that the V10Gy < 90%, V20Gy < 75% and V30Gy < 60% could be met in 100% of cases where VMAT margins were utilised (Table S1), suggesting that these objectives could be prioritised. The V40Gy < 37% was achieved in 83.3% of cases in our cohort; therefore, aiming to achieve this with a relatively low priority is feasible, until further prospective studies are available.

### *Bone* *marrow sparing planning*

Our study aimed to significantly reduce dose to the ABM from V10‐40Gy, whilst achieving clinically acceptable target and OAR doses when compared to plans where this was not considered.

#### Target and OAR doses

We found significant improvement in target coverage in BMS‐VMAT plans, except for the PTV D95, which was statistically significantly worse. The actual difference is −0.45Gy and would be clinically insignificant because all BMS‐VMAT plans met target objectives and were ensured to be comparable to the original plan. Therefore, this significant difference could be attributed to uneven comparison groups and the variation of planning margins in the BMS‐VMAT group. Significant improvement was also noted for all OAR structures in BMS plans. In comparison, Liang et al and the INTERTECC‐2 trial, which both utilised functional imaging, reported that there were no significant changes in PTV coverage.^14^, ^16^ Of our original cohort, 70% were planned conformally, and it has been reported that BMS‐VMAT is superior in terms of OAR doses and target coverage compared to conformal techniques.^15^ Compared with original VMAT plans, there were no significant differences in target or OAR coverage (except for the bladder V30Gy) in the BMS plans, consistent with the comparisons made in the INTERTECC‐2 trial and Liang et al studies.^14^, ^16^

#### ABM doses

Bone marrow sparing volumetric modulated arc therapy demonstrated significant reductions in ABM doses at all levels; however, only the ABM V10‐20Gy were significant in comparison with the original VMAT group. This is inconsistent with literature that has reported bone marrow doses were reduced at all levels, shown in Table 4.^13^, ^14^, ^16^, ^17^

**Table 4:** Bone marrow sparing studies in pelvic cancers.

Study	Cohort	V10Gy (%)		V20Gy (%)		V30Gy (%)		V40Gy (%)		Non‐BMS technique	Method of bone marrow delineation	
		Non‐BMS	BMS	Non‐BMS	BMS	Non‐BMS	BMS	Non‐BMS	BMS			
Our study	Cervix (*n* = 18) Vaginal (*n* = 2)	96.1	85	90.3	72.5	69.8	57.5	54.4	38.1	Conformal and VMAT	PET/CT	SUV ≥ the mean TBM SUV
INTERTECC‐2^14^	Cervix (*n* = 83)	N/A	82.6	N/A	63.5	N/A	45.7	N/A	22.2	IMRT and VMAT	PET/CT	
Liang et al.^16^	Cervix (*n* = 19) Anal (*n* = 12)	95	85	82	70	65	54	N/A	N/A	IMRT and VMAT	MRI + PET/CT	≥ Mean SUV on PET ≤ Mean fat fraction on MRI
Gupta et al.^13^	Cervix (*n* = 10)	99.5	96	92.1	81.6	77.1	66.7	53.5	46.8	VMAT	CT‐Only	Inner cavity
Chigurupalli et al.^17^	Cervix (*n* = 10)	85.4	78.1	72.4	65.9	57.5	52.1	37.1	35.6	VMAT	CT‐Only	Inner cavity
Mell et al.^15^	Cervix (*n* = 7)	97.3	76.5	92.7	57.5	59.9	46.1	48.9	33.7	Conformal	CT‐Only	External bony contours

4F‐box = 4 field box, BMS = bone marrow sparing, CT = computerised tomography, IMRT = intensity modulated radiation therapy, MRI = magnetic resonance imaging, N/A = not analysed in study, PET = positron emission tomography, SUV = standard uptake value, TBM = total bone marrow, VMAT = volumetric modulated arc therapy, Vx = x% of dose covering % of structure.

Despite the lack of significance of the V30‐40Gy in our study in comparison with the original VMAT group, ABM objective compliance was improved from 50% to 100% for the V30Gy, and 50% to 83.3% for the V40Gy. Furthermore, our actual dose reduction at the V30Gy is comparable to that reported by Liang et al., which was significant at all dose levels.^16^ Our small sample size and heterogeneous planning margins potentially limit our ability to reduce ABM doses further and the statistical power at these levels.

No other study has assessed ABM objective compliance rates for multiple ABM dose objectives. The ABM V10Gy < 90%, V20Gy < 75% and V30Gy < 60% are easily achievable and were compliant in 80‐95% of cases regardless of planning margin. Where VMAT margins are used, these could be met 100% of the time, and the V40Gy < 37% in 83.3% of cases. The V30<46.5% and V40<23% were unobtainable in our study, and therefore, it may be difficult to meet these constraints clinically. Our median values are comparable to Liang et al. (V30Gy = 54%), which would not achieve ideal constraints.^16^ In comparison, the INTERTECC‐2 trial achieved lower ABM doses than what was achieved in this study, indicating that a proportion of these patients could meet ideal ABM objectives. In their study, they utilised smaller target planning margins, reducing overlap and improving dose sparing of the ABM at these levels. Similarly, they considered their ABM objectives as hard constraints, whereas meeting these objectives was not essential in our study to prioritise target coverage.^14^

The key unanswered question from this study is how this will translate clinically. Most research in BMS techniques is retrospective, and the INTERTECC‐2 trial is currently the only prospective trial that has investigated the toxicity outcomes of BMS techniques for cervical cancer. They found that rates of grade 3+ leukopenia were reduced from 41.7% to 25.7%, and any grade 3+ HT from 43.8% to 31.4%, and chemotherapy compliance was improved in the BMS arm.^14^ A prospective study in anal and cervical cancer by Liang et al. also noted that all patients were able to tolerate treatment without hospitalisation, transfusions or treatment interruptions.^16^ Further prospective research is clearly needed to investigate the clinical impacts and identify patients who would significantly benefit from using this technique.

### Limitations

Our small sample size potentially impacts the statistical power of our tests. As a retrospective study, we cannot determine the clinical impact or outcomes, and this would need to be tested further in a prospective setting. To meet our sample size requirements (*n* = 20) in our original group, a range of techniques (i.e. protocols and prescriptions) and treatment sites were included that can alter the significance and reduce the ability to spare the ABM due to larger treatment margins. To minimise this limitation, the original group was separated based on plan technique; however, this resulted in uneven groups (conformal = 14, VMAT = 6 and BMS = 20) with varying prescriptions (conformal = 1 high dose, 5 low doses; VMAT = 4 high doses, 10 low doses), limiting statistical interpretation.

## Conclusion

This study demonstrated that BMS‐VMAT is a feasible method to reduce dose to ABM. In comparison with non‐BMS VMAT plans, significant sparing was found at ABM V10‐20Gy but not V30‐40Gy. Yet, despite lack of significance, ABM objective compliance improved, and our actual dose outcomes are comparable to those of other prospective studies. Future prospective research is needed to establish a protocol to translate this technique clinically.

## Funding

MB received a $5000 scholarship from the South Western Sydney Local Health District to partake this project.

## Conflict of Interest

The author declares no conflict of interest.

## Supporting information


**Table S1**. Additional target and OAR dose metrics.
**Table S2**. Plan characteristics and ABM objective compliance.

## Data Availability

Data are available upon request.

## References

[1] Australian Institute of Health and Welfare & Cancer Australia . Gynaecological cancers in Australia: an overview. Cancer Series No. 70. Cat. No. CAN 66. AIHW, Canberra, 2012.

[2] Monk K , Krishnansu TS , Koh WJ . Multimodality therapy for locally advanced cervical carcinoma: state of the art and future directions. J Clin Oncol 2007; 25: 2952–65. 17617527 10.1200/JCO.2007.10.8324

[3] Samant B , Lau B , Choan E , Le T , Tam T . Primary vaginal cancer treated with concurrent chemoradiation using Cis‐platinum. Int J Radiat Oncol Biol Phys 2007; 69: 746–50. 17512130 10.1016/j.ijrobp.2007.04.015

[4] Zhu H , Zakeri K , Vaida F , et al. Longitudinal study of acute haematologic toxicity in cervical cancer patients treated with chemoradiotherapy. J Med Imaging Radiat Oncol 2015; 59: 386–93. 25828068 10.1111/1754-9485.12297 PMC6373449

[5] Mazeron R , Castelnau‐Marchand P , Dumas I , et al. Impact of treatment time and dose escalation on local control in locally advanced cervical cancer treated by chemoradiation and image‐guided pulsed‐dose rate adaptive brachytherapy. Radiother Oncol 2015; 114: 257–63. 25497872 10.1016/j.radonc.2014.11.045

[6] Pötter R , Tanderup K , Kirisits C , et al. The EMBRACE II study: The outcome and prospect of two decades of evolution within the GEC‐ESTRO GYN working group and the EMBRACE studies. Clin Transl Radiation Oncol 2018; 9: 48–60. 10.1016/j.ctro.2018.01.001 PMC5862686 29594251

[7] Mell LK , Kochanski JD , Roeske JC , et al. Dosimetric predictors of acute hematologic toxicity in cervical cancer patients treated with concurrent cisplatin and intensity‐modulated pelvic radiotherapy. Int J Radiat Oncol Biol Phys 2006; 66: 1356–65. 16757127 10.1016/j.ijrobp.2006.03.018

[8] Wang Y , Tian Y , Tang Y , et al. A Phase II prospective nonrandomized trial of magnetic resonance imaging‐guided hematopoietic bone marrow‐sparing radiotherapy for gastric cancer patients with concurrent chemotherapy. Onco Targets Ther 2016; 9: 2701–7. 27217780 10.2147/OTT.S91586 PMC4863589

[9] David J , Yong Y , Blas K , Hendifar A , Kabolizadeh P , Tuli R . 18F‐FDG PET predicts hematologic toxicity in patients with locally advanced anal cancer treated with chemoradiation. Adv Radiat Oncol 2019; 4: 613–22. 31681863 10.1016/j.adro.2019.06.005 PMC6817719

[10] Wang SB , Jia‐Pei L , Kai‐Jian L , et al. The volume of 99mTc sulfur colloid SPET‐defined active bone marrow can predict grade 3 or higher acute hematologic toxicity in locally advanced cervical cancer patients who receive chemoradiotherapy. Cancer Med 2019; 8: 7219–26. 10.1002/cam4.2601 31621208 PMC6885884

[11] Rose BS , Aydogan B , Liang Y , et al. Normal tissue complication probability modeling of acute hematologic toxicity in cervical cancer patients treated with chemoradiotherapy. Int J Radiat Oncol Biol Phys 2011; 79: 800–7. 20400238 10.1016/j.ijrobp.2009.11.010 PMC2907446

[12] Klopp AH , Moughan J , Portelance L , et al. Hematologic toxicity in RTOG 0418: a phase 2 study of postoperative IMRT for gynecologic cancer. Int J Radiat Oncol Biol Phys 2013; 86: 83–90. 23582248 10.1016/j.ijrobp.2013.01.017 PMC4572833

[13] Gupta N , Chandra P , Chakrabarty K , Giri U , Patel A , Choudhary S . Potential advantages of bone marrow sparing IMRT in cancer cervix: a dosimetric evaluation. J Clin Diagn 2019; 13: 1–5.

[14] Mell LK , Sirak I , Wei L , et al. Bone marrow‐sparing intensity modulated radiation therapy with concurrent cisplatin for stage IB‐IVA cervical cancer: an International Multicenter Phase II CLINICAL TRIAL (INTERTECC‐2). Int J Radiat Oncol Biol Phys 2017; 97: 536–45. 28126303 10.1016/j.ijrobp.2016.11.027

[15] Mell LK , Tiryaki H , Ahn KH , Mundt A , Roeske JC , Aydogan B . Dosimetric comparison of bone marrow‐sparing intensity‐modulated radiotherapy versus conventional techniques for treatment of cervical cancer. Int J Radiat Oncol Biol Phys 2008; 71: 1504–10. 18640499 10.1016/j.ijrobp.2008.04.046

[16] Liang Y , Bydder M , Yashar CM , et al. Prospective study of functional bone marrow‐sparing intensity modulated radiation therapy with concurrent chemotherapy for pelvic malignancies. Int J Radiat Oncol Biol Phys 2013; 85: 406–14. 22687195 10.1016/j.ijrobp.2012.04.044

[17] Chigurupalli K , Vashistha A , Patel D , Purohit R , Peter S , Bhandari M . Retrospective dosimetric analysis of bone marrow sparing vs non bone marrow sparing image guided volumetric modulated arc therapy in intact carcinoma cervix patients. Curr Med Res Opin 2019; 2: 288–92.

[18] Rose BS , Liang Y , Lau SK , et al. Correlation between radiation dose to 18F‐FDG‐PET defined active bone marrow subregions and acute hematologic toxicity in cervical cancer patients treated with chemoradiotherapy. Int J Radiat Oncol Biol Phys 2012; 83: 1185–91. 22270171 10.1016/j.ijrobp.2011.09.048

[19] Elicin O , Callaway S , Prior JO , Bourhis J , Ozsahin M , Herrera FH . [18F]FDG‐PET standard uptake value as a metabolic predictor of bone marrow response to radiation: impact on acute and late hematological toxicity in cervical cancer patients treated with chemoradiation Therapy. Int J Radiat Oncol Biol Phys 2013; 90: 1099–107. 10.1016/j.ijrobp.2014.08.017 25442041

[20] Gay HA , Barthold HJ , O’Meara E , et al. Pelvic normal tissue contouring guidelines for radiation therapy: a radiation therapy oncology group consensus panel atlas. Int J Radiat Oncol Biol Phys 2012; 83: e353–e62. 22483697 10.1016/j.ijrobp.2012.01.023 PMC3904368

[21] Cancer Therapy Evaluation Program CTCfAE, Version 5.0, DCTD, NCI, NIH, DHHS, November 27. Available from: https://ctep.cancer.gov/protocolDevelopment/electronic_applications/docs/CTCAE_v5_Quick_Reference_5x7.pdf

[22] Rose BS , Jee KW , Niemierko A , et al. Irradiation of FDG‐PET–defined active bone marrow subregions and acute hematologic toxicity in anal cancer patients undergoing chemoradiation. Int J Radiat Oncol Biol Phys 2016; 94: 747–54. 26972647 10.1016/j.ijrobp.2015.12.006

[23] Special considerations regarding absorbed‐dose and dose–volume prescribing and reporting in IMRT. J ICRU. 2010; 10: 27–40. 10.1093/jicru/ndq008 24173325

[24] McGuire SM , Bhatia SK , et al. Using FLT PET to quantify and reduce hematologic toxicity due to chemoradiation therapy for pelvis cancer patients. Int J Radiat Oncol Biol Phys 2016; 96: 229–39. 10.1016/j.ijrobp.2016.04.009 PMC4982822 27319286

