# Supplementary material for: Feasibility of bone marrow sparing volumetric modulated arc therapy to spare active bone marrow in cervical and vaginal cancer patients: a retrospective dosimetric analysis
Source: J Med Radiat Sci. 2021 Jul 19;68(4):379–88. doi: 10.1002/jmrs.529 (PMC8655883; doi:10.1002/jmrs.529)
Supplement: Supplementary file 1 — Table S1. Additional target and OAR dose metrics. Table S2. Plan characteristics and ABM objective compliance. [file JMRS-68-379-s001.docx]

***Supporting Information Table S1: Additional Target and OAR Dose Metrics***

| **Structures** | | **Planning Median** | | | | **Significance** | | |
| --- | --- | --- | --- | --- | --- | --- | --- | --- |
|  |  | **Original** | | | **BMS** |  |  |  |
| **Name** | **Constraint** | **Conformal (n=14)**  **(IQR)** | **VMAT**  **(n=6)**  **(IQR)** | **Total**  **(n=20)**  **(IQR)** | **VMAT**  **(n =20)**  **(IQR)** | **Original Combined vs BMS** | **Original Conformal vs BMS** | **Original VMAT vs BMS** |
| **ITV** | V95% | 100%  (0.00) | 100%  (0.00) | 100%  (0.00) | 100%  (0.00) | P = 1.000 | P = 1.000 | P = 1.000 |
| **CTV** | V95% | 100%  (0.00) | 100%  (0.00) | 100%  (0.00) | 100%  (0.00) | P = 1.000 | P = 1.000 | P = 1.000 |
| **PTV** | V95% | 99.86%  (0.30) | 100%  (0.08) | 99.89%  (0.23) | 99.99%  (0.10) | P = 0.332 | P = 0.118 | P = 1.000 |
| **Small Bowel** | Point Max | 105.59%  (1.72) | 104.12%  (2.05) | 104.89%  (2.17) | 105.26%  (1.83) | P = 0.824 | P = 1.000 | P = 0.688 |
| **Rectum** | Point Max | 104.56%  (0.70) | 104.72%  (2.20) | 104.56%  (1.08) | 104.77%  (1.37) | P = 0.824 | P = 1.000 | P = 0.688 |
| **Bladder** | Point Max | 103.50%  (1.92) | 105.39%  (1.53) | 104.47%  (2.19) | 104.99%  (0.99) | P = 0.824 | P = 0.180 | P = 0.219 |
| **LT Iliac Crest** | V30Gy | 66.46%  (23.25) | 40.23%  (8.30) | 58.70%  (25.70) | 46.20%  (11.85) | **P < 0.001*** | **P < 0.001*** | P = 0.688 |
|  | V40Gy | 46.40%  (8.63) | 21.72%  (8.48) | 42.57%  (22.90) | 27.56%  (5.50) | **P = 0.003*** | **P < 0.001*** | P = 1.000 |
|  | V50Gy | 0.00%  (26.04) | 0.00%  (0.30) | 0.00%  (16.67) | 0.00%  (7.53) | P = 0.219 | P = 0.063 | P = 1.000 |
| **RT Iliac Crest** | V30Gy | 62.68%  (25.37) | 41.66%  (21.90) | 59.70%  (21.55) | 47.10%  (10.12) | **P = 0.003*** | **P < 0.001*** | P = 1.000 |
|  | V40Gy | 45.22%  (8.48) | 25.09%  (15.47) | 41.66%  (14.74) | 27.54%  (7.43) | **P < 0.001*** | **P < 0.001*** | P = 0.219 |
|  | V50Gy | 0.00%  (22.42) | 0.00%  (0.71) | 0.00  (16.55) | 0.00  (6.92) | P = 0.219 | P = 0.063 | P = 1.000 |
| **LT Femoral Head** | V30Gy | 23.06%  (21.21) | 13.16%  (20.13) | 20.84%  (18.56) | 11.77%  (7.37) | **P < 0.001*** | **P < 0.001*** | P = 0.219 |
|  | Point Max | 46.93Gy  (5.25) | 44.44Gy  (2.56) | 46.18Gy  (4.54) | 45.99  (5.80) | P = 0.263 | P = 0.180 | P = 1.000 |
| **RT Femoral Head** | V30Gy | 22.95%  (12.45) | 16.58%  (18.36) | 21.23%  (12.84) | 11.69%  (6.16) | **P < 0.001*** | **P < 0.001*** | P = 0.688 |
|  | Point Max | 46.41Gy  (5.03) | 45.17Gy  (2.57) | 46.28Gy  (1.75) | 46.35Gy  (2.27) | P = 0.824 | P = 0.791 | P = 1.000 |
| **External Genitalia** | V20Gy | 4.81%  (41.64) | 0.26%  (82.34) | 2.29% (44.93) | 0.00%  (5.99) | **P = 0.012*** | **P = 0.008*** | P = 1.000 |
|  | V30Gy | 0.00%  (2.02) | 0.00%  (29.26) | 0.00%  (2.11) | 0.00%  (0.13) | **P = 0.031*** | P = 0.125 | P = 0.500 |
|  | V40Gy | 0.00%  (0.00) | 0.00%  (2.84) | 0.00%  (0.00) | 0.00%  (0.00) | P = 0.625 | P = 0.500 | P = 1.000 |
| **Cauda Equina/ Spinal Cord** | Point Max | 45.87Gy  (6.80) | 36.78Gy  (12.78) | 44.08Gy  (8.93) | 38.23Gy  (12.10) | **P < 0.001*** | **P < 0.001*** | P = 0.219 |
| **LT Kidney** | Mean | 0.31Gy  (0.69) | 0.18Gy  (0.41) | 0.24Gy  (0.58) | 0.29Gy  (0.65) | **P = 0.035*** | **P = 0.006*** | P = 1.000 |
| **RT Kidney** | Mean | 0.48Gy  (1.30) | 0.06Gy  (0.45) | 0.31Gy  (1.00) | 0.49Gy  (0.93) | P = 0.424 | P = 0.388 | P = 1.000 |

* Statistically significant

*IQR = inter-quartile range. VMAT = volumetric modulated arc therapy. BMS = bone marrow sparing. ITV = internal target volume. CTV = clinical target volume. PTV = planning target volume. RT = right. LT = left. Vx = x% of dose covering % of structure*

***Supporting Information Table S2: Plan Characteristics and ABM Objective Compliance***

| **ID** | **Site** | **Prescription** | **Original Technique** | **PTV Volume (cc)** | **ABM Volume (cc)** | **Volume of ABM in PTV (cc)**  **(% of ABM)** | **Compliance** | | | | | | | |
| --- | --- | --- | --- | --- | --- | --- | --- | --- | --- | --- | --- | --- | --- | --- |
|  |  |  |  |  |  |  | **BMS** | | | | **Original** | | | |
|  |  |  |  |  |  |  | **V10** | **V20** | **V30** | **V40** | **V10** | **V20** | **V30** | **V40** |
| 01 | Cervix | 45 | VMAT | 1555.51 | 492.54 | 122.71  (24.77%) | ✓ | ✓ | A | A | ✕ | ✓ | A | ✕ |
| 02 | Endocervix | 45 | VMAT | 1223.25 | 550.52 | 62.11(11.28%) | ✓ | ✓ | A | A | ✕ | ✕ | ✕ | ✕ |
| 03 | Cervix | 45 | VMAT (EMBRACE) | 1474.08 | 432.47 | 52.83  (12.22%) | ✓ | ✓ | A | A | ✕ | ✕ | ✕ | A |
| 04 | Vagina | 45 | VMAT | 1956.7 | 543.68 | 136.66  (25.14%) | ✓ | ✓ | A | A | ✕ | ✕ | A | A |
| 05^†^ | Endocervix | LD = 45  HD = 50.4 | VMAT | LD = 2137.31  HD = 107.68 | 543.51 | LD = 173.38  (31.9%)  HD = 5.07  (0.93%) | ✓ | ✓ | A | ✕ | ✕ | ✕ | ✕ | ✕ |
| 06 | Cervix | 50.4 | Conformal | 1731.7 | 401.65 | 156.3  (38.91%) | ✓ | ✓ | A | ✕ | ✕ | ✕ | ✕ | ✕ |
| 07 | Cervix | 45 | Conformal | 1397.55 | 545 | 89.84  (16.48%) | ✓ | ✓ | A | A | ✕ | ✕ | A | ✕ |
| 08 | Cervix | 45 | Conformal | 1616.18 | 520.11 | 124.7  (23.98%) | ✓ | ✓ | A | ✕ | ✕ | ✕ | ✕ | ✕ |
| 09 | Cervix | 45 | Conformal | 1473.68 | 445.35 | 120.18  (26.99%) | ✓ | ✓ | ✕ | ✕ | ✕ | ✕ | ✕ | ✕ |
| 10 | Endocervix | 45 | Conformal | 1654.6 | 539.22 | 103.95  (19.28%) | ✓ | ✓ | A | A | ✕ | ✕ | ✕ | ✕ |
| 11 | Cervix | 45 | Conformal | 1997.23 | 497.64 | 120.19  (25.7%) | ✓ | ✓ | A | ✕ | ✕ | ✕ | ✕ | ✕ |
| 12 | Cervix | 45 | Conformal | 1869.9 | 564.71 | 122.93  (21.77%) | ✓ | ✕ | A | ✕ | ✕ | ✕ | ✕ | ✕ |
| 13 | Vagina | 45 | VMAT | 1242.01 | 616.46 | 133.56  (21.67%) | ✓ | ✓ | A | A | ✓ | ✓ | A | A |
| 14 | Cervix | 45 | Conformal | 1845.5 | 635.9 | 113.96  (17.92%) | ✓ | ✓ | A | A | ✕ | ✕ | ✕ | ✕ |
| 15 | Cervix | 50.4 | Conformal | 1455.66 | 431.3 | 147.55(34.21%) | ✕ | ✕ | ✕ | ✕ | ✕ | ✕ | ✕ | ✕ |
| 16 | Cervix | 45 | Conformal | 2094.69 | 610.47 | 125.21  (20.51%) | ✓ | ✓ | A | ✕ | ✕ | ✕ | ✕ | ✕ |
| 17 | Cervix | 45 | Conformal | 1919.04 | 516.09 | 122.74  (23.78%) | ✓ | ✓ | A | ✕ | ✕ | ✕ | ✕ | ✕ |
| 18 | Cervix | 50.4 | Conformal | 1847.34 | 482.15 | 101.89  (21.13%) | ✓ | ✕ | A | ✕ | ✕ | ✕ | ✕ | ✕ |
| 19 | Cervix | 50.4 | Conformal | 2048.56 | 570.4 | 165.4  (28.99%) | ✓ | ✓ | A | ✕ | ✕ | ✕ | ✕ | ✕ |
| 20 | Cervix | 50.4 | Conformal | 1294.34 | 419.73 | 100.27  (23.89%) | ✓ | ✕ | A | ✕ | ✕ | ✕ | ✕ | ✕ |
| TOTAL COMPLIANCE (%) (n=20) | | | | | | | 95 | 80 | 90 | 40 | 5 | 10 | 10 | 15 |
| Original VMAT Compliance (%) (n = 6) ^‡^ | | | | | | | 100 | 100 | 100 | 83.33 | 16.67 | 33.33 | 50 | 50 |
| Original Conformal Compliance (%) (n=14) ^§^ | | | | | | | 92.86 | 71.43 | 85.71 | 21.43 | 0 | 0 | 7.15 | 0 |

† Patient was treated with multidose levels. LD = low dose PTV at 45Gy, and HD = high dose PTV at 50.4Gy

‡ In the BMS group, this is when the original technique was VMAT

§ In the BMS group, this is when the original technique was conformal.

✓ = ideal constraint met. A = acceptable constraint met. ✕ = doesn’t meet constraint.

*PTV = planning target volume. ABM = active bone marrow. BMS = bone marrow sparing. VMAT = volumetric modulated arc therapy. LD = low dose level. HD = high dose level.*
